# Risk factors for immune-related adverse events associated with anti-PD-1 pembrolizumab

**DOI:** 10.1038/s41598-019-50574-6

**Published:** 2019-10-01

**Authors:** Yeonghee Eun, In Young Kim, Jong-Mu Sun, Jeeyun Lee, Hoon-Suk Cha, Eun-Mi Koh, Hyungjin Kim, Jaejoon Lee

**Affiliations:** 1 Department of Medicine, Samsung Medical Center, Sungkyunkwan University School of Medicine, Seoul, Republic of Korea; 2 Division of Rheumatology, Department of Medicine, National Police Hospital, Seoul, Republic of Korea; 3 Division of Hematology-Oncology, Department of Medicine, Samsung Medical Center, Sungkyunkwan University School of Medicine, Seoul, Republic of Korea

**Keywords:** Cancer therapy, Cancer immunotherapy

## Abstract

We investigated risk factors for immune-related adverse events (irAEs) in patients treated with anti-programmed cell death protein1 antibody pembrolizumab. A retrospective medical record review was performed to identify all patients who received at least one dose of pembrolizumab at Samsung Medical Center, Seoul, Korea between June 2015 and December 2017. Three hundred and ninety-one patients were included in the study. Data were collected on baseline characteristics, treatment details, and adverse events. Univariate and multivariate logistic regression models were used to identify risk factors for irAEs. Sixty-seven (17.1%) patients experienced clinically significant irAEs; most commonly dermatologic disorders, followed by pneumonitis, musculoskeletal disorders, and endocrine disorders. Fourteen patients (3.6%) experienced serious irAEs (grade ≥ 3). Most common serious irAEs were pneumonitis (2.3%). Four deaths were associated with irAEs, all of which were due to pneumonitis. In multivariate regression analysis, a higher body mass index (BMI) and multiple cycles of pembrolizumab were associated with higher risk of irAEs (BMI: odds ratio [OR] 1.08, 95% confidence interval [CI] 1.01–1.16; pembrolizumab cycle: OR 1.15, 95% CI 1.08–1.22). A derived neutrophil-lymphocyte ratio (dNLR) greater than 3 at baseline was correlated with low risk of irAEs (OR 0.37, 95% CI 0.17–0.81). Our study demonstrated that an elevated BMI and higher number of cycles of pembrolizumab were associated with an increased risk of irAEs in patients treated with pembrolizumab. Additionally, increased dNLR at baseline was negatively correlated with the risk of developing irAEs.

## Introduction

Immune checkpoints are regulatory molecules of the immune system and play an important role in maintaining immune homeostasis and self-tolerance^1^. The first immune checkpoints that were identified include cytotoxic T-lymphocyte protein-4 (CTLA-4) and programmed cell death protein-1 (PD-1)^2^. CTLA-4 is expressed on the surface of T cells, binds to B7-1 (CD80) or B7-2 (CD86) molecules on antigen-presenting cells, and functions as a negative regulator of T cells^3^. PD-1 also has a negative effect on T cell activity through interactions with its ligands, including programmed death ligand-1 (PD-L1) and programmed death ligand-2 (PD-L2)^4^. Unlike CTLA-4, PD-1 is not only found on T cells but is also broadly expressed on many immunologic cells, including B cells and natural killer cells^5,6^. In healthy individuals, the surface expression of both CTLA-4 and PD-1 is tightly and dynamically regulated^7,8^.

During the development of cancer, malignant cells inhibit the immune response by activating immune checkpoints. Previous studies have shown that PD-L1 is expressed in a wide range of cancers^9–11^. In the tumor microenvironment, PD-L1 expressed by cancerous cells interacts with PD-1 on the surface of T cells to inhibit effector function of T cells. In addition, a number of studies have demonstrated that high tumor expression of PD-L1 is significantly correlated with poor prognosis of carcinoma^12,13^. These studies suggest that there is a therapeutic effect of PD-1 signaling pathway blockade in cancer.

Recent clinical trials have revealed that several anti-PD-1 and anti-PD-L1 immune checkpoint inhibitors (ICIs) are effective in a variety of cancers, such as melanoma, non-small cell lung carcinoma, renal cell carcinoma, and head and neck cancer^14–16^. Additional clinical trials are currently underway to expand the indication for ICIs. To date, The U.S. Food and Drug Administration (FDA) has approved three anti-PD-1 antibodies, nivolumab, pembrolizumab, and cemiplimab, and three anti-PD-L1 inhibitors, atezolizumab, avelumab, and durvalumab, for the treatments of different types of cancer.

As the use of ICIs increases, the adverse events related to this class of drugs have become an important issue. ICIs have a different toxicity profile than conventional cytotoxic chemotherapy. The side effects associated with the increased activity of the immune system by ICIs, known as immune-related adverse events (irAEs), can affect multiple organs of the body including skin, gastrointestinal tract, endocrine system, liver, lung, nervous systems, and musculoskeletal systems. Studies have shown that up to 80% of patients receiving ICIs experience adverse events (AEs)^17–20^.

Although many reports related to irAEs have been recently published, few studies have investigated risk factors associated with irAEs. A systematic review and meta-analysis, for example, has shown distinct patterns of irAEs according to the ICI class (CTLA-4 or PD-1/PD-L1) or tumor type (melanoma or non-melanoma). However, in these studies, only the types of drugs were investigated for the risk factors of irAEs^21–26^. To our knowledge, data on additional factors that predict the occurrence of irAEs are lacking. Herein, by retrospective medical record review, we analyzed irAEs in patients treated with pembrolizumab at Samsung Medical Center, Seoul, Korea between June 2015 and December 2017, to identify risk factors associated with irAEs.

## Patients and Methods

### Patients

All patients aged 18 years and older who had received at least one dose of pembrolizumab at Samsung Medical Center, Seoul, Korea from June 2015 to December 2017 were included in this study. The exclusion criteria included patients who received pembrolizumab in combination with other ICIs or other therapeutic agents including conventional chemotherapeutic agents and targeted therapy, and patients who lacked follow up after one dose of pembrolizumab.

### Collection of data

Data from the time pembrolizumab was first prescribed until the time of switching medication, the time of death, or the end of the study period were collected through retrospective medical record review. At the time of starting pembrolizumab, demographic data including sex, age, body weight, height, BMI, cancer type, and laboratory test results were obtained. BMI was categorized into four groups according to the proposed classification in adult Asian population presented by World Health Organization; Underweight, BMI < 18.5 kg/m^2^; normal, 18.5 kg/m^2^ ≤ BMI < 23 kg/m^2^; overweight, 23 kg/m^2^ ≤ BMI < 25 kg/m^2^; obese, BMI ≥25 kg/m^2^ ^27^. A derived neutrophil to lymphocyte count (dNLR) was calculated using the formula: absolute neutrophil count/(white blood cell count – absolute neutrophil count)^28^. Collected data included details of pembrolizumab therapy (e.g., dose, cycle) and drug-related AEs and irAEs (e.g., occurrence, grade, type, treatment and progress). AEs were graded using the Common Terminology Criteria for Adverse Events v4.0. The grading of irAEs was determined by treating physicians (hematologists and oncologists). Patients suspected of having irAEs were reviewed through a chart review, and only those patients deemed highly likely to have irAEs were included in the study.

### Statistical analysis

Student’s t-test, chi-square, and linear by linear association test were used to compare baseline characteristics of patients with and without irAEs. We performed univariate and multivariate logistic regression analyses to identify variables associated with irAE development. All statistical analyses were conducted using SPSS, version 24 (SPSS Inc., Chicago, IL, USA). The present study protocol conformed to the ethical guidelines of the 1975 Declaration of Helsinki, as revised in 1983, and was approved by the Institutional Review Board of Samsung Medical Center. The need for informed consent was waived owing to the retrospective nature of the study.

## Results

A total 391 patients were included in the study, of which 63.2% were male. The median age was 60 years (18–95 years), and the median follow-up time was 48 days (1–794 days). The primary malignancies included in the study were lung cancer, melanoma, lymphoma, and others (Table 1). The median dose per cycle of pembrolizumab was 167 mg (100–240 mg), and the median number of cycles was 2 (1–36). Table 1 shows a comparison of baseline characteristics between patients who experienced irAEs and those who did not.

**Table 1:** Comparison of baseline characteristics between patients with and without immune-related adverse events*.

	Patients with irAEs (n = 67)	Patients without irAEs (n = 324)	
**Age, years**	59 (52–66)	60 (52–69)	0.309
**Sex, male**	45 (67.2)	202 (62.3)	0.457
**BMI, kg/m** ^**2**^	23.5 (20.8–25.8)	22.5 (20.2–24.5)	0.040
**BMI category** ^**†**^			**0.211**
Underweight	4 (6.0)	39 (12.0)	
Normal	26 (38.8)	146 (45.1)	
Overweight	18 (26.9)	74 (22.8)	
Obese	19 (28.4)	65 (20.1)	
**Cancer type**			**0.091**
Lung cancer	32 (47.8)	179 (55.2)	
Melanoma	21 (31.3)	53 (16.4)	
Lymphoma	8 (11.9)	45 (13.9)	
Gastric cancer	1 (1.5)	15 (4.6)	
Urothelial cancer	2 (3.0)	6 (1.9)	
Others	3 (4.5)	26 (8.0)	
**Pembrolizuamb therapy**			
Dose/cycle, mg	133 (100–200)	185.5 (100–200)	0.708
Number of cycles	4 (2–8)	2 (1–4)	<0.001
Cumulative dose, mg	600 (300–1200)	400 (200–600)	0.001
**Baseline laboratory finding**			
WBC, cells/mm^3^	6000 (4860–7710)	7280 (5400–9865)	0.012
ANC, cells/mm^3^	3680 (2600–5900)	4700 (3160–7343)	0.010
dNLR	2.16 (1.10–2.40)	3.13 (1.40–3.60)	0.005
dNLR category			**0.001**
<3	58 (86.6)	216 (66.7)	
≥3	9 (13.4)	108 (33.3)	
Eosinophil count, cells/mm^3^	92 (21–210)	99 (28–189)	0.998
Albumin, g/dL	4.1 (3.8–4.4)	4.0 (3.5–4.4)	0.110

^*^Data are presented as median with interquartile range or number (%). irAEs, immune-related adverse events; BMI, body mass index; WBC, white blood cell count; ANC, absolute neutrophil count; dNLR, derived neutrophil/lymphocyte ratio.

^†^BMI is categorized based on the proposed classification of weight by BMI in adult Asians^27^.

Underweight, BMI < 18.5 kg/m^2^; normal, 18.5 ≤ BMI < 23; overweight, 23 ≤ BMI < 25; obese, BMI ≥ 25.

The incidence of any grade and grade 3 or higher AEs were 27.1% and 4.1% respectively. Sixty-seven patients experienced 88 irAEs; 14 of whom experienced 16 grade 3 or higher irAEs. Dermatological disorders were the most commonly reported irAEs followed by pneumonitis musculoskeletal disorders and endocrinopathies (Table 2). Table 3 summarizes the serious irAEs, including pneumonitis. There were 4 deaths associated with irAEs, all of which were due to pneumonitis. The majority of patients who experienced severe irAEs were treated with steroids (Table 3).

**Table 2:** Clinically significant immune-related adverse events*.

	All grade	Grade ≥ 3
Dermatologic	49 (55.7)	3 (3.4)
Pruritis	24 (27.3)	—
Rash	23 (26.1)	3 (3.4)
Vitiligo	2 (2.3)	—
Musculoskeletal	12 (13.6)	2 (2.3)
Myalgia	5 (5.7)	—
Myositis	1 (1.1)	1 (1.1)
Myasthenia gravis	1 (1.1)	1 (1.1)
Arthralgia	3 (3.4)	—
Arthritis	2 (2.3)	—
Pulmonary	11 (12.5)	9 (10.2)
Endocrinopathy	7 (8.0)	—
Gastrointestinal	5 (5.7)	1 (1.1)
Diarrhea	4 (4.6)	—
Enterocolitis	1 (1.1)	1 (1.1)
Hepatic	3 (3.4)	1 (1.1)
Dry mouth	1 (1.1)	—
Total	88 (100.0)	16 (18.2)

*All data are presented as number of cases (%).

**Table 3:** Summary of cases with serious immune-related adverse events*.

Patient	Age (years)	Sex	BMI (kg/m^2^)	Cancer	No. of Cycles	irAEs	Grade	Treatment	Prognosis
1	20	M	16.5	Hodgkin lymphoma	5	Pneumonitis	3	High dose steroid	Resolution Pembrolizumab resumed
2	55	F	23.5	Vulvar sarcomatoid carcinoma	3	Pneumonitis	5	High dose steroid	Death due to pneumonitis
3	62	M	26.3	NSCLC	2	Pneumonitis	3	High dose steroid	Resolution Pembrolizumab discontinued
4	66	M	26.5	NSCLC	4	Pneumonitis	3	High dose steroid	Resoultion
						Skin rash	2	Antihistamine	Pembrolizumab discontinued
						Pruritis	1		
5	57	M	22.9	NSCLC	2	Pneumonitis	3	High dose steroid	Resolution Pembrolizumab discontinued
6	57	M	23.8	NSCLC	1	Pneumonitis	3	High dose steroid	Resolution Pembrolizumab discontinued
7	52	M	27.9	NSCLC	2	Pneumonitis	5	High dose steroid Mechanical ventilation	Death due to pneumonitis
8	56	M	21.2	NSCLC	2	Pneumonitis	5	High dose steroid	Death due to pneumonitis
9	59	M	20.7	NSCLC	1	Pneumonitis	5	High dose steroid	Death due to combined infection
10	51	M	33.0	NSCLC	1	Hepatitis	3	High dose steroid	Improvement Death due to respiratory failure
11	80	F	22.5	Melanoma	2	Myositis	3	High dose steroid	Resolution
						Myasthenia gravis	4	Pyridostigmine IV immunoglobulin Mechanical ventilation	Pembrolizumab discontinued
12	60	M	23.5	NSCLC	8	Skin rash	3	Topical steroid	Resolution
						Pruritis	2	Low dose steroid Antihistamine	Pembrolizumab discontinued
13	64	F	21.8	Bladder cancer	1	Skin rash	3	High dose steroid Antihistamine	Resolution Pembrolizumab resumed
14	61	M	33.3	NSCLC	1	Skin rash	3	Antihistamine	Resolution Ongoing pembrolizumab therapy

*BMI, body mass index; irAEs, immune-related adverse events; NSCLC, non-small cell lung cancer.

^†^Number of cycles of pembrolizumab.

Univariate binary logistic regression analysis was performed to assess risk factors for irAEs (Table 4). A higher BMI was associated with irAEs. If the BMI increases by 1 kg/m^2^, the risk of irAEs increases by 9% (odds ratio [OR] = 1.09, 95% confidence interval [CI] 1.02–1.17, p = 0.012). The risk of irAEs in melanoma was 2.2 times higher than in lung cancer (OR = 2.22, 95% CI 1.18–4.16, p = 0.013). Among the baseline laboratory data, a dNLR greater than 3 showed a negative correlation with the risk of developing irAEs (OR = 0.31, 95% CI 0.15–0.65, p = 0.002). In addition, the analysis revealed that number of cycles and cumulative dose of pembrolizumab were both risk factors for irAEs (p < 0.001 for both; Supplementary Fig. S1).

**Table 4:** Univariate binary logistic regression analysis to determine risk factors for immune- related adverse events*.

	Odds ratio	95% CI	*p*
Age	0.99	0.97–1.01	0.494
**Sex**			
Male	1 (Reference)	—	—
Female	0.81	0.46–1.41	0.457
BMI	1.09	1.02–1.17	0.012
Underweight l	0.58	0.19–1.75	0.330
Norma	1 (Reference)		
Overweight	1.34	0.70–2.65	0.357
Obese	1.64	0.85–3.18	0.141
**Cancer type**			
Lung cancer	1 (Reference)	—	—
Melanoma	2.22	1.18–4.16	0.013
Lymphoma	0.99	0.43–2.31	0.990
Gastric cancer	0.37	0.05–2.92	0.348
Urothelial cancer	1.87	0.36–9.65	0.458
Others	0.65	0.18–2.26	0.493
**Pembrolizumab**			
Number of cycles	1.15	1.09–1.22	<0.001
Cumulative dose	1.00	1.00–1.00	<0.001
**Baseline laboratory finding**			
WBC	0.95	0.89–1.01	0.102
ANC	0.94	0.88–1.01	0.080
dNLR	0.831	0.71–0.97	0.016
<3	1 (Reference)	—	—
≥3	0.31	0.15–0.65	0.002
Eosinophil counts	1.00	1.00–1.00	0.639
Albumin	1.62	1.01–2.58	0.043

*CI, confidence interval; BMI, body mass index; WBC, white blood cell counts; ANC, Absolute neutrophil counts; dNLR, derived neutrophil/lymphocyte ratio.

Subsequently, variables included in the final multivariate regression model were selected according to their clinical relevance and statistical significance in a univariate analysis (cutoff: p = 0.20; Table 5). An elevated BMI and multiple cycles of pembrolizumab were associated with higher risk of irAEs in patients treated with pembrolizumab, with an OR of 1.08 (95% CI 1.01–1.16, p = 0.036; Supplementary Fig. S2) and 1.15 (95% CI 1.08–1.22, p < 0.001). We conducted additional analysis by dividing the cycle into two categories with a reference value of 2. When pembrolizumab was administered more than once, the risk of the occurrence of irAEs increased 2.4 times as compared with single dose only (OR 2.44 in multivariate models, 95% CI 1.04–5.70, p = 0.040). In contrast, the risk of irAEs was significantly lower in patients with a baseline dNLR of 3 or more than those with a baseline dNLR less than 3 (OR = 0.37, 95% CI 0.17–0.81, p = 0.012; Supplementary Table S1 and Supplementary Fig. S3).

**Table 5:** Multivariate logistic regression analysis of factors associated with immune-related adverse events*.

	Odds ratio	95% CI	*p*
BMI	1.08	1.01–1.16	0.036
Number of pembrolizumab cycle	1.15	1.08–1.22	<0.001
dNLR ≥ 3	0.37	0.17–0.81	0.012

*Adjusted for sex, age, cancer type, absolute neutrophil counts, and albumin. CI, confidence interval; BMI, body mass index; dNLR, derived neutrophil/lymphocyte ratio.

## Discussion

In this study, we analyzed demographic and lab test data, and data on irAEs occurring in patients treated with pembrolizumab, and identified risk factors associated with these irAEs. We found that the risk of irAEs increased by 9% when BMI increased by 1 kg/m^2^. In addition, patients who received more than one dose of pembrolizumab were at least twice as likely to develop irAEs than those with only one dose. Furthermore, high dNLR at baseline was negatively correlated with risk of irAEs.

The reported incidence of irAEs varies between previous studies, but as many as 80% of patients treated with ICIs experience AEs^29,30^. In our study population, irAEs occurred in 17% of patients. There are several reasons why fewer irAEs have been reported than expected. First, mild AEs that do not require substantive action are unlikely to be recorded in clinical practice. Due to the fact that the data analyzed in this study was collected from retrospective chart review, the incidence of side effects may be lower than other clinical trials where reporting of AEs are more thorough and rigorous. Second, the physicians’ lack of awareness of irAEs may have contributed to few reports of rheumatologic AEs such as arthralgia, myalgia, and sicca symptoms. Indeed, suboptimal reporting of irAEs has been demonstrated in a systematic review of clinical trials of ICIs^23^. Nevertheless, the clinically meaningful irAEs, especially those requiring intervention, thoroughly included in this study.

The most common irAEs in this study were dermatologic, followed by musculoskeletal, pulmonary, and endocrine disorders. The most common serious irAE was pneumonitis, with higher reported incidence compared to other studies^24,30,31^. A possible explanation for this result is that the incidence of pneumonitis may have been overestimated due to the fact that other conditions could have been mistaken for pneumonitis, such as infection, and were not filtered into the retrospective chart review.

Recently, predictors of irAEs have been performed mainly on patients with melanoma treated with anti-CTLA-4 antibody ipilimumab. In those studies, an increase in serum interleukin (IL)-17 or an increase in eosinophil count was associated with irAEs^32,33^.

Other studies have shown that immune cell infiltration of bowel mucosa in the early stage of treatment was associated with gastrointestinal toxicity, and that diversification of T-cell repertoire was associated with irAEs^34,35^. Several possible baseline risk factors for irAEs, including personal and family history of autoimmune diseases, tumoral infiltration, opportunistic pathogens, co-medications, and professional exposures, have been proposed, but there is little evidence to support the association of these factors with the development of irAEs^36^.

A previous investigation of 84 patients with malignant melanoma treated with ipilimumab showed an increased risk of high-grade AEs in patients with BMI >25 kg/m^2^ ^37^. Consistent with the above finding, the results of the present study also indicated that a higher BMI was associated with an elevated risk for developing irAEs. Possible explanations for these results are that obesity is an inflammatory condition, and may play a role in promoting inflammation involved in the development of irAEs. Leptin, an adipokines secreted by adipocytes, plays a proinflammatory role by stimulating the production of IL-1, IL-6, IL-12, and tumor necrosis factor-α (TNF-α), promoting T cell proliferation, and inhibiting regulatory T cell proliferation^38^. Elevated levels of inflammatory cytokines in the sera of patients with irAEs and the improvement of some irAEs with anti-TNF treatment suggest that these cytokines are involved in the development of irAEs^39,40^. Therefore, it is reasonable to speculate that proinflammatory cytokines, which are secreted in association with adipose tissue inflammation, likely promote the development of irAEs.

Neutrophil to lymphocyte ratio (NLR) and dNLR are simple and cost-effective markers that can be obtained using standard blood tests in clinical practice. High NLR is associated with poor outcome in many types of cancer^41^. In recent studies of patients with non-small cell lung cancer and malignant melanoma treated with ICIs, pretreatment dNLR greater than 3 was correlated with worse outcomes^42–44^. These results imply that tumor-induced neutrophil polarization and activation are associated with ICIs treatment failure^45^. Our study showed that higher dNLR was linked to lower risk of irAEs. Prior studies have demonstrated that overall and progression-free survival were longer in patients who developed irAEs than those who did not develop irAEs^46–50^. Considering this positive correlation between irAEs and a response to ICIs, it can be assumed that neutrophils, which are altered in cancer, negatively impact the outcome of cancer immunotherapy or the development of irAEs.

The relationship between the occurrence of irAEs and the number of cycles of pembrolizumab can also be interpreted in the same context. In patients with irAEs, the treatment with ICIs might be more effective in terms of survival, which might lead to continued use of pembrolizumab. Another possibility is that the longer exposure to pembrolizumab increases the risk of developing irAEs. To test these two hypotheses, further investigations taking into account the treatment outcome and the time of occurrence of irAEs are needed.

There are some limitations to our study. First, the study was conducted by retrospective manner, and therefore information bias might have been included. Also, because it was carried out only in one center in South Korea, generalizability could be limited in applying this results to other countries and races. Second, some variables failed to demonstrate statistical significance due to the small number of cases. A multi-center study that include larger number of cases will be useful in confirming the relationship between BMI and irAEs. Third, the outcome of this study did not include assessment of treatment outcomes such as overall survival and progression-free survival. Future research is needed to establish the association of treatment outcome with irAEs and their association with biomarkers. However, previous studies have focused primarily on improved progression-free survival or overall survival in patients with irAEs, so this study has the advantage of identifying risk factors for the occurrence of irAEs.

In conclusion, the risk for irAEs was increased with an elevated BMI and higher number of pembrolizumab cycles. A dNLR greater than 3 was associated with lower risk for irAEs. These results may be useful for predicting and monitoring patients at high risk of developing irAEs. Additional studies are warranted to elucidate the mechanism by which obesity is involved the treatment of ICIs and how the tumor-induced neutrophil polarization affects the cancer immunotherapy.

## Supplementary information

 Supplementary Figures and Table

## Data Availability

The datasets generated during and/or analyzed during the current study are available from the corresponding author upon reasonable requests.
