# Supplementary material for: Risk factors for immune-related adverse events associated with anti-PD-1 pembrolizumab
Source: Sci Rep. 2019 Oct 1;9:14039. doi: 10.1038/s41598-019-50574-6 (PMC6773778; doi:10.1038/s41598-019-50574-6)

Risk factors for immune-related adverse events associated with anti-PD-1 pembrolizumab

Yeonghee Eun[1], In Young Kim[2], Jong-Mu Sun[3], Jeeyun Lee[3], Hoon-Suk Cha[1], Eun-Mi Koh[1], Hyungjin Kim[1,*], and Jaejoon Lee[1,*]

[1]Division of Rheumatology, [3]Division of Hematology, Department of Medicine, Samsung Medical Center, Sungkyunkwan University School of Medicine, Seoul, Republic of Korea

[2]Division of Rheumatology, Department of Medicine, National Police Hospital, Seoul, Republic of Korea.

[*]Drs. Hyungjin Kim and Jaejoon Lee contributed equally to this work.

[*]Correspondence to: Division of Rheumatology, Department of Medicine, Samsung Medical Center, Sungkyunkwan University School of Medicine, 81 Irwon-ro, Gangnam-gu, Seoul 06351, Republic of Korea.

E-mail address: Chandler.kim@samsung.com (H. Kim), jaejoonlee.lee@samsung.com (J.Lee).

Supplementary Table S1. Univiariate and multivariate analysis of effect of continuous and categorized derived neutrophil-to-lymphocyte ratio values for immune-related adverse events

	Univariate analysis			Multivariate analysis		
**Variables**	**Odds ratio**	**95% CI**	***p***	**Odds ratio**	**95% CI**	***p***
NLR as continuous variable	0.83	0.71-0.97	0.016	0.86	0.75-1.00	0.053
NLR as dichotomous variable						
Cut-off ≥2	0.48	0.28-0.83	0.009	0.61	0.35-1.09	0.094
Cut-off ≥3	0.31	0.15-0.65	0.002	0.37	0.17-0.81	0.012
Cut-off ≥4	0.50	0.23-1.10	0.084			
Cut-off ≥5	0.52	0.21-1.25	0.143			
Cut-off ≥6	0.46	0.16-1.35	0.157			

Supplementary Figure S1. The relation between irAEs grade and pembrolizumab cycle

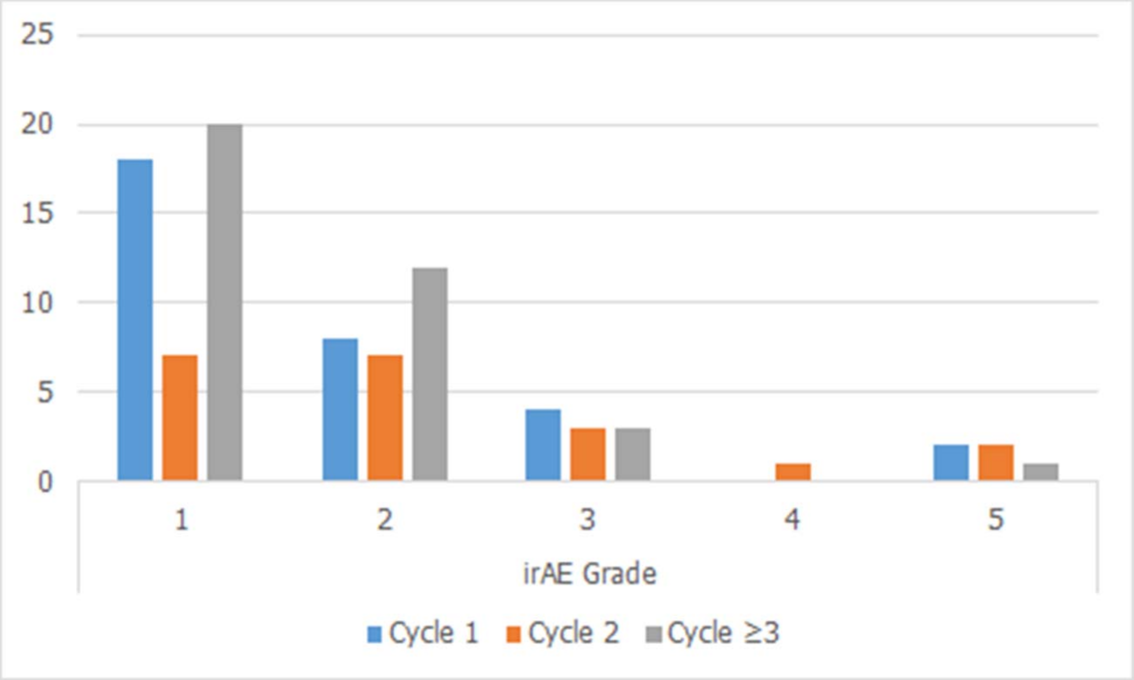

Supplementary Figure S2. The occurrence of irAEs according to BMI category

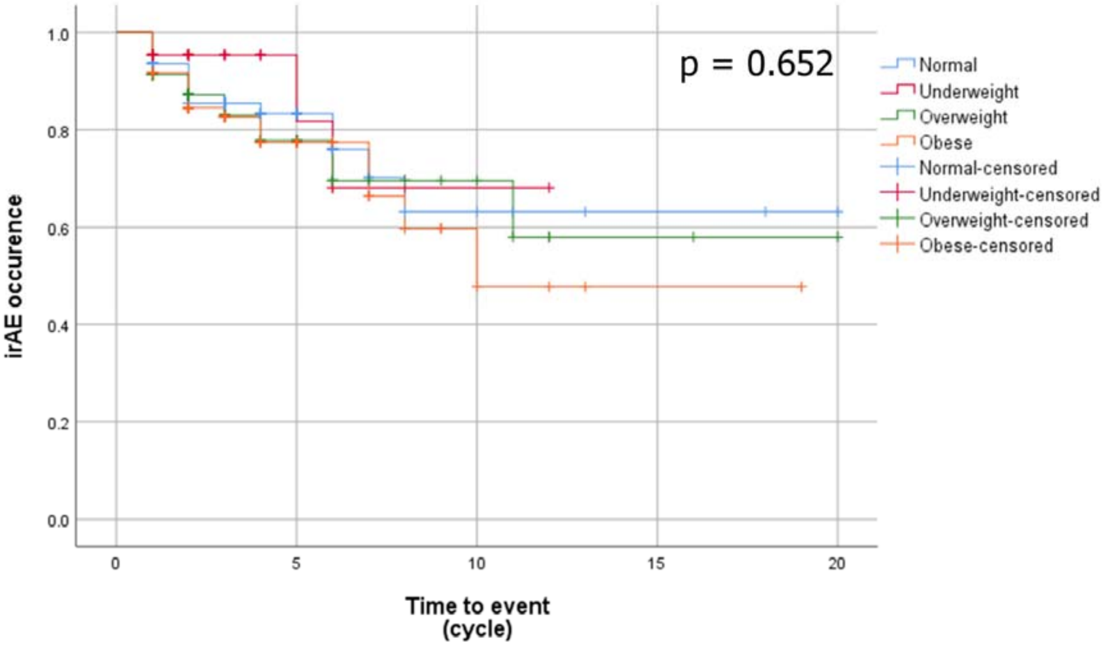

Supplementary Figure S3. The occurrence of irAEs according to dNLR

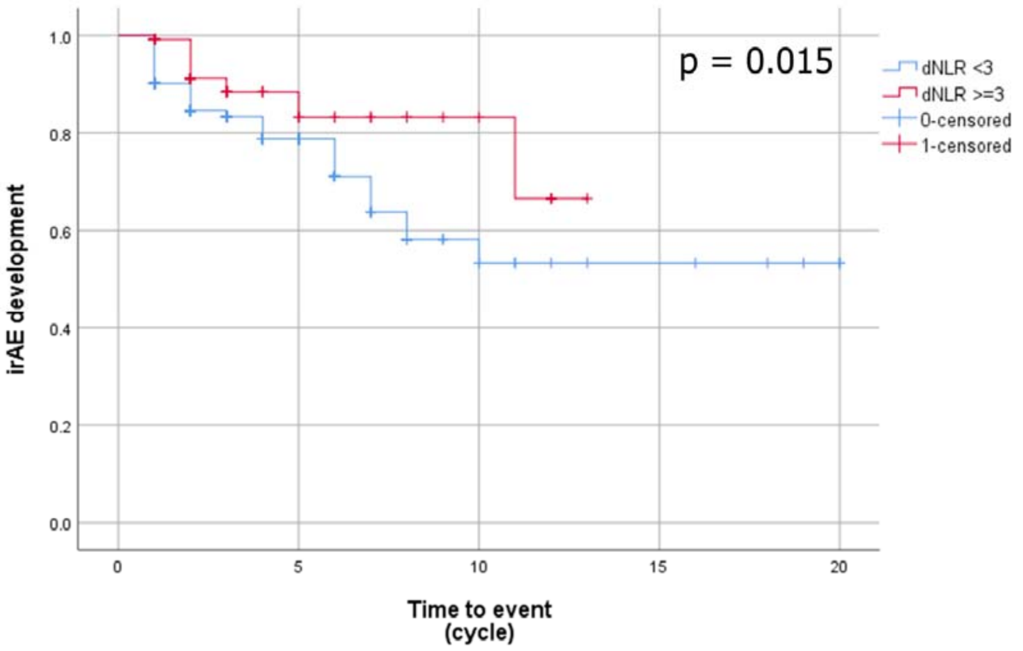

Supplement: Supplementary file 1 — Supplementary Figures and Table [file 41598_2019_50574_MOESM1_ESM.pdf]
